# Complete Genome Sequence of a Mesophilic Obligately Chemolithoautotrophic Hydrogen-Oxidizing Bacterium, Hydrogenovibrio marinus MH-110

**DOI:** 10.1128/MRA.01132-19

**Published:** 2019-10-17

**Authors:** Hiroyuki Arai, Masaharu Ishii

**Affiliations:** a Department of Biotechnology, Graduate School of Agricultural and Life Sciences, The University of Tokyo, Bunkyo, Tokyo, Japan; b Collaborative Research Institute for Innovative Microbiology, The University of Tokyo, Bunkyo, Tokyo, Japan; Georgia Institute of Technology

## Abstract

Hydrogenovibrio marinus is a mesophilic, obligately chemolithoautotrophic, and hydrogen-oxidizing bacterium that uses three different RubisCOs at different carbon dioxide tensions. Here, we report its complete genome sequence, which is 2,491,293 bp long, with an average GC content of 44.1%.

## ANNOUNCEMENT

Hydrogenovibrio marinus MH-110 (JCM7688 [https://www.jcm.riken.jp/cgi-bin/jcm/jcm_number?JCM=7688], DSM11271 [https://www.dsmz.de/collection/catalogue/details/culture/DSM-11271]) is an aerobic, mesophilic, obligately chemolithoautotrophic, and hydrogen-oxidizing bacterium isolated from seawater of the Shonan Coast, Japan (1). It was isolated with hydrogen as an energy source. The bacterium fixes carbon dioxide using three different RubisCOs encoded by different operons (2–5). Carboxysome is produced at low carbon dioxide tensions. Strain MH-110 has an oxygen-tolerant [NiFe]-hydrogenase and accumulates glycogen under nitrogen or magnesium starvation or oxygen limitation (6–8). Two groups have independently reported a draft genome sequence of this strain (DSM11271 [https://www.dsmz.de/collection/catalogue/details/culture/DSM-11271]). However, the complete genome had not been determined (9, 10). We determined the complete genome sequence in order to facilitate further studies on the application of this bacterium to produce useful materials from carbon dioxide.

The whole genome of strain MH-110, maintained by our laboratory, was sequenced using a Roche 454 GS FLX instrument. Total DNA was isolated by a standard phenol-chloroform method from the cells, cultivated autotrophically in an inorganic medium with the gas phase consisting of H_2_, O_2_, and CO_2_ (75:15:10, vol/vol/vol) (1). We constructed the 8-kb mate pair library following the standard procedure (11). The data processing, quality control, and assembly of contigs and scaffolds were performed using the Roche GS FLX software v.2.8 and GS De Novo Assembler v.2.6. Default software parameters were used. The paired-end sequencing yielded 137,935,170 bases from 294,573 reads, with an average read length of 248 bp. After removing adaptors and low-quality reads, the reads were assembled into 54 contigs of >500 bp. The genome coverage was 55.4-fold, and the average contig length and *N*_50_ size were 45,539 bp and 139,857 bp, respectively. The Q40 plus bases showed 99.92% reliability. Four scaffolds were generated from 36 contigs using the paired-end information. The average scaffold length and *N*_50_ size were 622,477 bp and 2,478,578 bp, respectively. Gaps between the contigs or scaffolds were closed by sequencing DNA fragments amplified by PCR from genomic DNA with an ABI 3730xl DNA analyzer (Applied Biosystems). *Ex Taq* or *LA Taq* DNA polymerase with GC buffer II (TaKaRa) was used for the PCR amplification. The custom primers used for PCR and Sanger sequencing were designed near the ends of the contigs. The genes were annotated using the DDBJ Fast Annotation and Submission Tool (DFAST) (https://dfast.nig.ac.jp/) with ARAGORN to predict tRNA genes.

The complete genome of strain MH-110 comprises a circular chromosome of 2,491,293 bp with 44.1% GC content. The genome contains 2,322 predicted protein-coding genes, 3 sets of the rRNA genes, and 43 tRNA genes. The scaffolds of approximately 100 kb, presumably derived from a plasmid, reported in the previous draft genome sequences (9, 10) were not identified in the genome of our laboratory strain. A 133.4-kb region between the rRNA gene clusters was inverted compared with the previous draft genome sequences.

### Data availability.

The complete genome sequence of *H. marinus* MH-110 has been deposited in DDBJ under accession number AP020335. The raw sequence data have been deposited in the DDBJ Sequence Read Archive under accession number DRA008898.
